# Application of Functionalized Graphene Oxide Based Biosensors for Health Monitoring: Simple Graphene Derivatives to 3D Printed Platforms

**DOI:** 10.3390/bios11100384

**Published:** 2021-10-10

**Authors:** Agnivo Gosai, Kamil Reza Khondakar, Xiao Ma, Md. Azahar Ali

**Affiliations:** 1 Corning Inc., Science & Technology, Painted Post, NY 14870, USA; agnivo2007@gmail.com; 2 Department of Electrical and Computer Engineering, Florida International University, Miami, FL 33174, USA; kamilreza@gmail.com; 3 Department of Biomedical Engineering, New York University, Brooklyn, NY 11201, USA; 4 Department of Mechanical Engineering, Carnegie Mellon University, Pittsburgh, PA 15235, USA

**Keywords:** graphene, functionalized graphene, graphene oxide, biosensor, 3D Printing

## Abstract

Biosensors hold great potential for revolutionizing personalized medicine and environmental monitoring. Their construction is the key factor which depends on either manufacturing techniques or robust sensing materials to improve efficacy of the device. Functional graphene is an attractive choice for transducing material due to its various advantages in interfacing with biorecognition elements. Graphene and its derivatives such as graphene oxide (GO) are thus being used extensively for biosensors for monitoring of diseases. In addition, graphene can be patterned to a variety of structures and is incorporated into biosensor devices such as microfluidic devices and electrochemical and plasmonic sensors. Among biosensing materials, GO is gaining much attention due to its easy synthesis process and patternable features, high functionality, and high electron transfer properties with a large surface area leading to sensitive point-of-use applications. Considering demand and recent challenges, this perspective review is an attempt to describe state-of-the-art biosensors based on functional graphene. Special emphasis is given to elucidating the mechanism of sensing while discussing different applications. Further, we describe the future prospects of functional GO-based biosensors for health care and environmental monitoring with a focus on additive manufacturing such as 3D printing.

## 1. Introduction

Biosensors detect various analytes (deoxyribonucleic acid, ribonucleic acid, proteins, cells, and pathogens) in biofluids such as serum, blood, and urine, and trace harmful microorganisms/chemicals/agrochemical waste in the environment (air, water, soil, etc.) [1,2]. Detection of these analytes is very important for disease screening and treatment, and in environmental monitoring. Traditionally circulating biomarkers (proteins, cells, and nucleic acids), pathogens (viruses, bacteria, yeast, etc.), and toxic chemicals are being investigated using enzyme linked immunosorbent assay (ELISA), lateral flow assays, flow cytometry, DNA sequencing, reverse transcription-polymerase chain reaction (rt-PCR), and high-performance liquid chromatography (HPLC) [3,4]. Due to advancements in nanoscience/technology, researchers are focused on early detection of disease biomarkers and environmental testing of analytes for better healthcare management. Despite showing high sensitivity towards lower detection limits, these techniques still lack clinical sensitivity for early and rapid diagnosis of diseases. Next-generation equipment will be relying on early detection, portability, and quicker decision making for developing better healthcare monitoring systems. Researchers are exploring various next-generation two-dimensional nanomaterials such as (Ti_3_C_2_T_x_) MXenes, molybdenum disulfide (MoS_2_), and graphene to achieve ultra-sensitive biomedical sensors for point-of-care diagnostics and environmental monitoring with high accuracy [5,6].

Graphene has shown great promise in the development of biosensing devices and is of increasing interest for the development of ultra-sensitive biosensors due to its single-atom layer thickness, extremely high carrier mobility, unique electrical conductivity, and inherently low electrical noise [7]. Since the invention of graphene by Geim and coworkers in 2004, numerous formations of graphene such as graphene oxide (GO), reduced graphene oxide (rGO), and graphene quantum dots (GQD) have been applied in biomedical sensors including enzymatic biosensors, immunosensors, and DNA sensors for health monitoring and agriculture sensing [8,9]. This is because of its low-cost fabrication, larger surface area (2630 m^2^/g for single layer graphene), and the fact that it is easy to functionalize with biomolecules as functional graphene has large number of groups and high electron transfer properties. Owing to direct electron transfer and high electrocatalytic properties, functional graphene is in high demand for developing sensitive biosensors.

Among the graphene derivatives, GO-based transducers exhibit excellent unique characteristics that enable the development of ultrasensitive devices for detection of minute amount of target analytes (biomolecules, chemicals, toxic waste) due to their high surface area to volume ratio, high electron mobility, good water dispersibility, biocompatibility, and size controllability [10,11]. In addition, GO has been demonstrated as a promising nanomaterial in applications such as drug delivery, electronics, wearable devices, and bioimaging [12,13]. Its ease of surface modification using various methods to control the size of its nanosheets and its unique physical and chemical properties make GO an attractive material for biosensor fabrication as well as for designing active surfaces for patterning on solid supports such as gold, platinum, indium tin oxide, and silver electrode. The various groups present on GO nanosheets are epoxides (C–O–C), phenolic hydroxyl (–OH), and carboxylic (–COOH) and other carbonyl groups (C=O) which provide covalent modification/functionalization for covalent linkage of chemical bonds or functional groups onto the surface for biomolecule attachment. To fabricate optical sensors, GO can be processed in colloidal suspension and it is easily complexed with biomolecules to produce highly efficient long-range photoluminescence signals [10]. The properties of graphene and its derivatives have been explored for developing label-free optical sensors such as optical fiber-based sensors, surface plasmon resonance (SPR) sensing, and surface-enhanced Raman scattering for bioimaging, antibacterial activity, and drug delivery system [13,14]. Furthermore, GO has a high surface area to volume ratio that provides abundant active sites for direct coupling with the antibody resulting in improved system performance. Moreover, GO-based immunoassay has successfully demonstrated a much wider dynamic range and was found to be more sensitive than the same immunoassay using biotin–streptavidin functionalization [14].

Lately, microfluidic systems have paved a generic way for miniaturization, integration, automation, and parallelization of (bio-)chemical processes such as isolation/detection of analytes from biological fluids on a single chip [15]. Currently, integration of graphene with optofluidic and electrochemical microfluidic chips is of great interest in biomarker sensing owing to offering multiplex sensing with high sensitivity and militarization. Trau et al. developed a microfluidic platform that uses GO and gold-based biochips for sensitive cell and protein analysis in human serum samples [14]. GO containing negatively charged oxygenated functional groups at physiological pH, and the hexagonal aromatic graphene structure, promoted hydrogen bonding and electrostatic, hydrophobic van der Waals, and π–π interactions allowing it to interact with protein analytes [16]. Their GO-based chip successfully demonstrated the higher capture yields and lower detection limits for multiple biomarkers spiked into serum, which had not been reported earlier. With functionalized GO on the gold surface as an effective sensing platform, they were able to capture and characterize single cells (10–20 cells per mL) and proteins (10 fg/mL) in human serum as an early disease detection system.

Another problem in clinical diagnosis is disease heterogeneity i.e., single marker detection is not enough in clinical settings. For example, there are reports about various mutations existing in the environment of the COVID-19 viruses requiring more robust diagnostic tool to capture all the variants of single virus type. Similarly in cancer treatment, instead of detecting individual analytes, it might be possible to characterize the molecular composition of a tumor indirectly, by sampling the blood and searching for alterations in the serum [17].Thus, detection of multiple biomarkers such as proteins, DNA/RNA, exosomes, and cells in the serum and blood sample of a cancer patient might provide more information about the disease stage and improve disease diagnosis, aiding the patient’s recovery. GO and its composites have the capability of detecting and analysis of multiple biomarkers in various platforms for point-of-care (POC) application. Campbell et al. demonstrated GO as a novel multifunctional platform for therapeutic delivery, biological imaging, and cancer sensing [13]. Similarly, three different proteins thrombin (TB), prostate specific antigen (PSA), and hemagglutinin (HA) were detected using both DNA and RNA aptamers immobilized on the GO surface to show the multiplicity capacity of GO-based aptasensors [18].

In addition to biomedicine, GO-based sensors have been applied in agriculture monitoring such as measurement of soil moisture and nutrient sensing, and as natural bio-imaging agents with photoluminescent potassium-doped GO from agricultural waste used for developing stable fluorescence probes for bio-imaging applications. Functionalized GO/iron (GO-Fe) composite was also utilized as a fertilizer to supply phosphate ions to soil for better agricultural product development [19,20,21]. In all-solid-state ion-selective sensors, GO acted as ion-to-electron transducing layer that replaced the internal-filling reference solution between the conductor layer and ion-selective membrane. For example, rGO combined with polypyrrole was used as an ion-to-electron transducing layer in an all-soli-state ion-selective sensor for nitrate detection in soils [22]. In this sensor, introduction of rGO exhibited long term stability of the sensor, negligible potential drift (0.67 ± 0.05 mV/h), higher Nernstian slope (56.2 ± 0.2 mV/decade), low detection limit (10–5.2 ± 0.1 M), wider linear range (10^−5^–10^−1^ M), and shorter detection time (≤15 s). This indicates that the application of functional graphene can be potentially used for agriculture monitoring for precision and sustainable farming.

**Figure 1:**
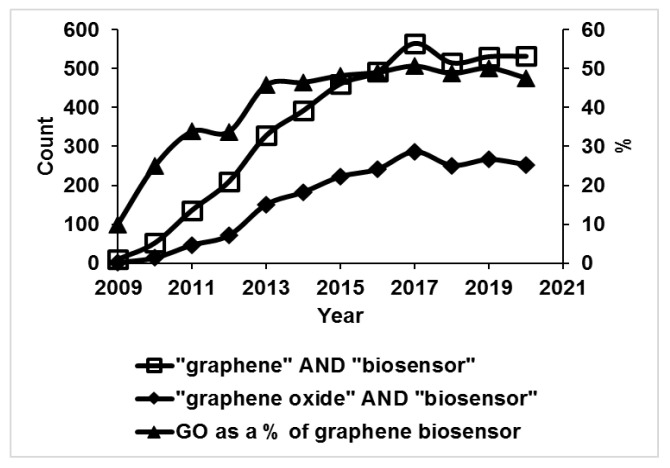
The chart shows the number of documents indexed in Scopus which have the following keyword combinations: “graphene” AND “biosensor”, “graphene oxide” AND “biosensor”, over the time span from 2009 to 2020. In 2009, there was one biosensor document with the keyword “graphene oxide” against 10 with the word “graphene”. The curve with triangular markers shows the percentage of biosensor articles containing “graphene oxide” for all articles containing “graphene” as a keyword, which indicates that during the last 5 years, these have been constant at around 48–50%. This suggests that GO is a dominant material for graphene-based biosensors, and it has been discussed in almost 50% of the biosensor articles on graphene.

**Figure 2:**
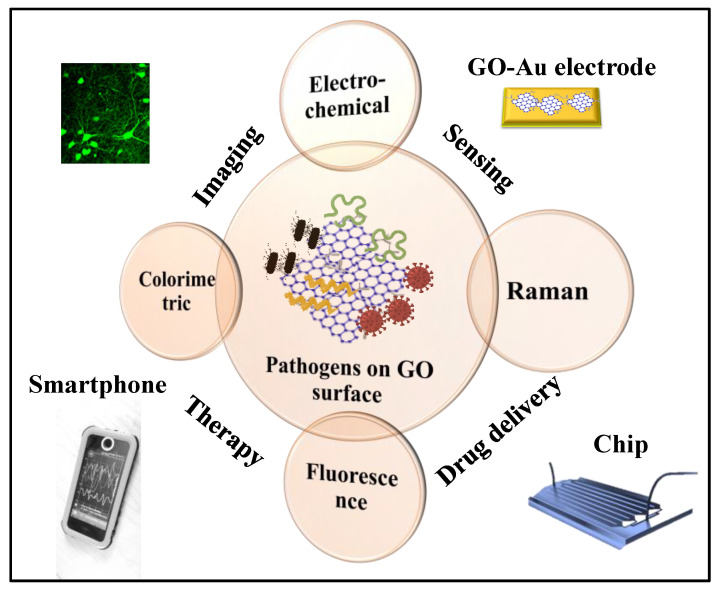
The application of GO in different fields including biosensors for detection of pathogens such as viruses and bacteria is presented in the schematic.

In this article, we critically review the application of graphene and functionalized graphene such as GO towards health monitoring. We have emphasized different sensing modalities based on functionalized graphene and its derivatives for the detection of biomarkers and chemicals. Specially, we cover examples of enzymatic biosensors, immunosensors, DNA sensors, and pathogenic biomarkers. Further, we critically discuss and summarize the future of biosensor technology based on graphene using the industry 4.0 manufacturing outlook that focuses on additive manufacturing (i.e., 3D printing). 3D printing enables the potential impact of constructing prototyping and developing production/commercial quality platforms in multidisciplinary areas such as electronics, biotechnology, aerospace and defense, and chemical engineering [23]. Figure 1 shows the number of biosensing applications per year for graphene and graphene-based material (data from Scopus) in the last decade and it may be seen that roughly 50% of graphene-based biosensors have use GO in the last 5 years. Although there are numerous applications of graphene oxide (which are detailed in Figure 2) we have also found that functional graphene and GO are a most exciting material for manufacturing of biosensors to monitor disease biomarkers. 

## 2. Functionalized Graphene Oxide for Enzymatic Biosensors 

**Figure 3:**
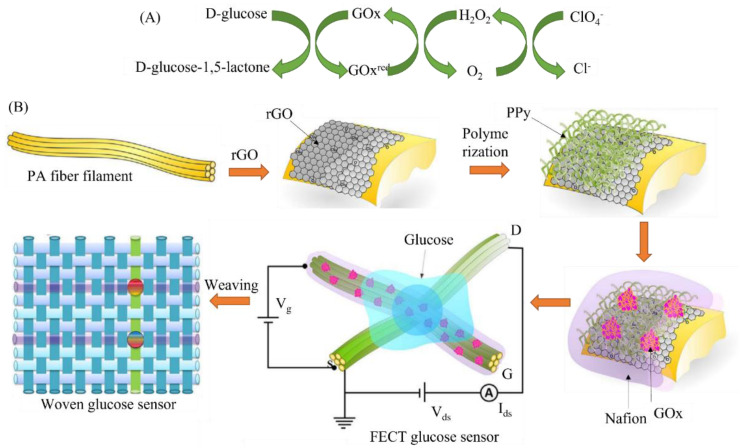
(**A**) Biochemical reaction shows glucose sensing using glucose-oxidase-based (GOx) polypyrrole (PPy) and reduced graphene oxide (rGO) [27] (**B**) A transistor-based glucose sensor. In this sensor, GOx was immobilized on PPy nanowires and rGO surface and further coated with Nafion. Nafion was used to improve selectivity of the sensor. Reprinted with permission from ref. [27] Copyright 2017 Elsevier.

In enzymatic biosensors, enzymes have been incorporated as bioreaction elements which allow catalytic biochemical reactions with specific target biomolecules. However, the main concerns are the enzyme functionalization and stability on a given transducing layer. GO has been repeatedly used to develop enzymatic biosensors and some of the novel reports are described in this section with a focus on the mechanism of detection. Zhou et al. [24] reported the performance enhancement of a zinc oxide (ZnO)-nanorod-based enzymatic glucose sensor with rGO introduced between the ZnO nanorods and indium tin oxide (ITO) electrode and then stimulated under UV irradiation. The electrochemical characterization indicated that the rGO not only facilitated electron transfer through the ZnO nanorods to the ITO electrode but also inhibited the fast recombination of the photo-generated electrons and holes. Ultra-violet (UV) irradiation stimulates holes in the valence band of the ZnO nanorods (Figure 3A,B), as oxidants enhance the catalytic activity of the glucose oxidase (GOx) towards glucose [25,26]. The rGO increased the sensitivity of the ZnO-nanorod-based glucose sensor 1.6-fold and decreased the detection limit 2.3-fold. The sensor also works on serum samples. Together with the rGO, UV irradiation further increased the sensitivity 1.7-fold and but diminished the detection limit 2-fold. Wang et al. [27] demonstrated an electrochemical transistor based on polypyrrole (PPy) nanowires and rGO for glucose sensing (Figure 3). The biochemical reaction is shown in Figure 3A [27]. Figure 3B shows the schematic presentation of sensor construction using a composite matrix of PPy and rGO [27]. The composite in the sensor not only holds glucose oxidase (enzyme) but also helps in the electron transfer from electrolyte to collector. In another, sensor it was found that rGO nanosheets promoted the growth and increase the number of PPy nanowires [28], and improved the electrical characteristics of fiber transistors such as on/off ratio, switch speed, and cycling stability. Glucose sensors based on these transistors exhibited excellent sensitivities, fast response times (~0.5 s), a wider linear range (1 nM to 5 μM), and a low limit-of-detection (LOD). Further, GO was modified with chitosan (a biocompatible material) that acted as a suitable transduction material for glucose sensing [29,30]. 

Covalent modification of GO is achieved by a standard carboxylic activation/amidation approach in the presence of available amino groups in chitosan. The composite GO−Ch was deposited on standard screen-printed electrodes (SPCE) by a drop-casting approach. Comparison between a chitosan—GO blend and pristine GO demonstrated the superior reliability and efficiency of the electrochemical response for glucose as a consequence of the high number of enzyme binding sites and of the partial reduction of GO during the carboxylic activation synthetic step. Zhou et al. [31] have reported a 1-aminopyrene-reduced graphene oxide (AP−rGOs) composite-based transducer that utilizes the direct electron transfer of laccase and its enzymatic oxidation of the analyte to detect the presence of toxic phenols. Laccase, the enzymatic receptor, is immobilized onto the AP−rGOs resulting in Lac/AP−rGOs through a glutaraldehyde-mediated cross-linker. Following treatment with chitosan, the Lac/AP−rGO/chitosan stock solution was dropped onto a GCE which could detect phenols in water samples and demonstrated a fast response time (<5 s), high stability (retained >97% activity after 7 days of storage), and an LOD of 2 and 7 μM for hydroquinone and catechol, respectively. Zhou and coworkers [32] have described a novel magnetic-controlled photoelectrochemical (PEC) sensing system developed for the sensitive detection of prostate-specific antigen (PSA) using reduced graphene oxide-functionalized bismuth ferrite (rGO-BiFeO_3_). The rGO-BiFeO_3_ acted as a photoactive material possessing accelerated charge transfer with improved visible light absorption. The biosensor involved an anchor DNA-conjugated magnetic bead (MB-aDNA), PSA aptamer/trigger DNA (Apt-tDNA), and two glucose-oxidase-labeled hairpins (H1-GOx and H2-GOx). When the target PSA reacted with the aptamer, initially the trigger DNA was released, which partially hybridized with the anchor DNA on the MB. Consequently, the unpaired trigger DNA on the MB allowed the opening of the hairpin DNA structures in sequence and this propagated a chain reaction of hybridization events between two alternating hairpins resulting in the formation of a long-necked double-helix with numerous GOx enzymes on it. Following this, hydrogen peroxide (H_2_O_2_) was generated as an enzymatic product and consumed the photo-excited electrons from rGO-BiFeO_3_ under visible light irradiation thereby enhancing the photocurrent. This intelligent combination of target-triggered hybridization chain reaction and enzyme-catalyzed photoelectric reaction enabled the biosensor to achieve an LOD of 0.31 pg/mL within the linear range of 0.001–100 ng/mL, in phosphate buffer saline (PBS) and showed remarkable performance in human serum. Huang et al. [33] have demonstrated a simple and ultrasensitive GO-based cholesterol biosensor incorporating gold nanoparticles (AuNPs). Cholesterol oxidase (CHOD), cholesterol esterase (CHER), and GO were immobilized onto the surface of AuNP-modified SPCE, which hydrolyzed the cholesterol to produce H_2_O_2._ This reduced the silver (Ag) ions in the cholesterol-containing silver nitrate (AgNO_3_) solution to metallic Ag. Anodic stripping voltammetry was used as the electrochemical technique and the reported LOD was 0.001 μg/mL. 

## 3. Graphene Oxide for Immunosensing

**Figure 4:**
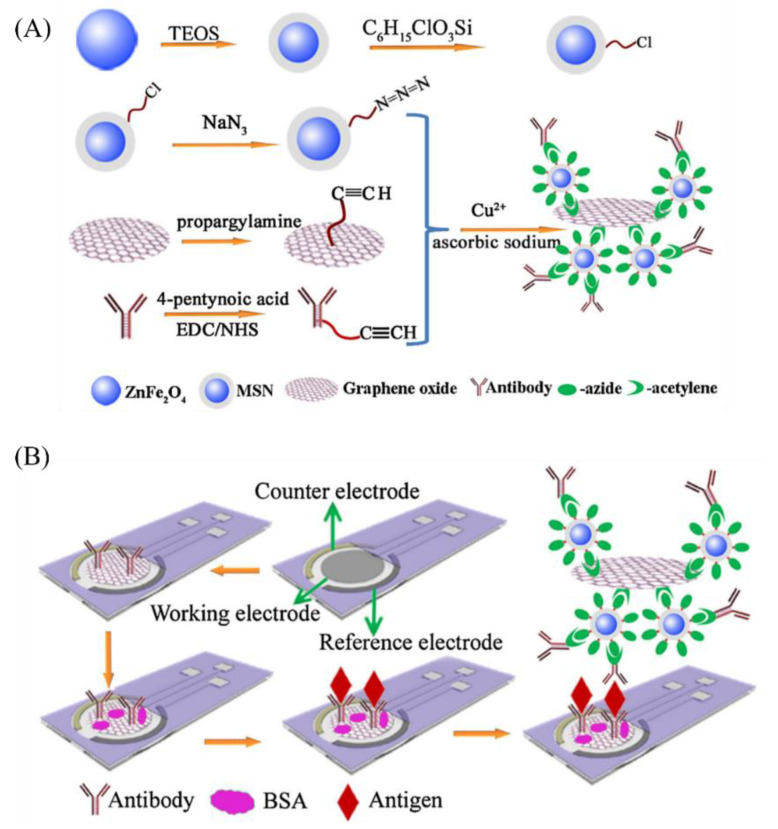
Graphene-oxide-based electrochemical immunosensor. In this sensor, graphene oxide was modified using click chemistry. (**A**) The magnetic silica nanoparticle/GO composite was synthesized via conjugation of azide-functionalized magnetic silica nanoparticles to acetylene-functionalized graphene oxide by click chemistry with a copper-catalyzed 1,3-dipolar cyclo-addition reaction. (**B**) The immunosensor for sensing cancer antigen 153 (CA 153) was fabricated following the sandwich method by immobilizing a monoclonal anti-CA 153 antibody on the GO attached on a screen-printed electrode, and H_2_O_2_-like magnetic silica nanoparticle/GO composites functioned as a signal label. Reprinted with permission from ref. [41] Copyright 2014 Elsevier.

Immunosensors [34,35] are a special class of biosensors, based on an antigen—antibody reaction, wherein the antibody or antigen is the receptor/recognition element. Graphene-based immunosensors have been described previously [36,37] and one of the first applications of GO in immunosensors is found in the work of Li et al. [38]. They used a transducer composed of graphene-polyaniline (GR−PANI) and carboxylated GO which increased the current response for the electrochemical sensor. The carboxylated GO facilitated the formation of horseradish peroxidase−GO−antibody (HRP-GO-Ab) conjugates, wherein the peroxidase increased the catalytic activity of hydrogen reduction, and the antibody was bound with the target 17β-estradiol. The sensor could detect estradiol in spiked samples of water and milk and performed better than conventional competitive electrochemical immunosensors [39] and aptamer-based electrochemical sensors [40]. A disposable immunosensor for the detection of cancer antigen 153 was reported by Ge et al. [41]. This used a sandwich method to immobilize monoclonal antibody on the GO-modified SPCE whose catalytic activity was augmented by peroxidase such as magnetic silica nanoparticles/graphene oxide labels (Figure 4). The authors reported detection in spiked human serum samples and excellent correlation with commercial electrochemiluminescent analyzer. A label-free immunosensor was reported by Ali et al. [42], wherein an aminated reduced-GO (rGO)-based electrode was constructed to immobilize anti-apolipoprotein B-100 that could bind with the target low-density-lipoprotein (LDL) cholesterol. The electroactive sites of rGO could promote increased heterogenous electron transport (HET) [43,44] and the high loading capacity of the antibody enabled a linear range of 5–120 mg/dL of LDL cholesterol in the electrochemical impedance spectroscopy (EIS)-based immunosensor. The sensor also demonstrated a fast reaction time of 250 s with a stability of 5 weeks. Singh et al. [45] reported the use of a AuNPs−rGO composite deposited onto an SPCE immunosensor for the electrochemical detection of the cardiac biomarker myoglobin. The AuNPs−rGO composite showed higher electrical conductivity compared to pristine rGO as the AuNPs formed an inter-penetrating network promoting electron conduction pathways. Additionally, the large surface area of nanostructured electrodes [46] and the oxygen-related defects in the rGO [47] increased the electrochemical response, enabling an eight-fold better LOD compared to ELISA. Another cardiac biomarker called cardiac troponin I (cTnI), which is used in the diagnosis of acute myocardial infarction (AMI), was detected using a porous GO nanostructure-based label-free impedimetric immunosensor as discussed by Kazemi et al. [48]. Ren et al. [49] have described the use of AuNP-decorated branched polyethylenimine-reduced GO in creating a competitive immunosensor for the detection of toxic melamine. The polyethylenimine acted as both a grafting agent and a reductant of GO and together with the AuNPs facilitated the increased electrochemical response. Thus, enormous possibilities of health monitoring devices introduced by realizing functionalized graphene have been reported by various researchers. 

## 4. DNA Sensing via Functionalized Graphene Oxide 

GO presents unique mechanical, optical, electrical, and chemical performance for construction of DNA-based biosensors. Among many sensing modalities, it serves as an effective acceptor of fluorescence resonance energy transfer (FRET) to quench the fluorescence of labeled DNA samples upon adsorption [50,51,52]. Such a property is prevalently applied in the DNA sensing. In addition, DNA sensing by GO typically possesses great precision, high selectivity, high sensitivity, and low detection limits at low cost.

**Figure 5:**
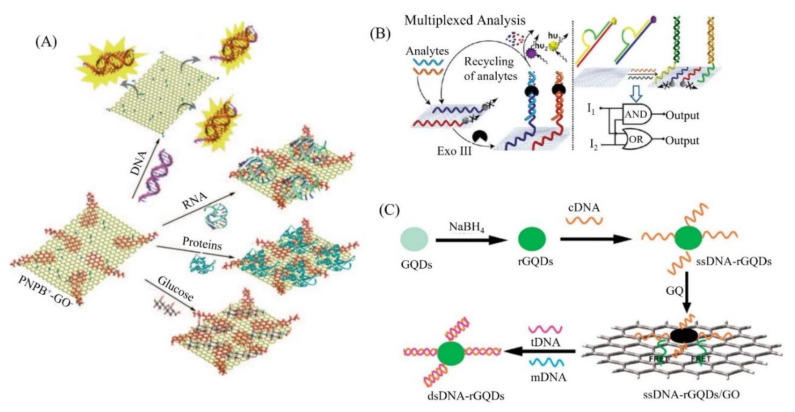
Construction of DNA-based biosensors using functionalized GO. (**A**) Specific DNA sensing by PNP^+^GO^−^ complex. Only DNA, but not RNA, proteins, or glucose can switch on the fluorescence due to the ion-exchange process. Reprinted with permission from ref. [54]. Copyright 2010 Wiley International Online. (**B**) GO is employed to amplify the multiplexed detection of DNA and aptamers, and implement the integration of DNA constructs to activate logic gate operations. Reprinted with permission from ref. [57]. Copyright 2012 ACS. (**C**) A universal fluorescence sensing platform for DNA detection based on FRET between GO and GQD. Reprinted with permission from ref. [58]. Copyright 2014 RSC.

Various types of GO biosensors for DNA detection have been developed over the past few years. Zhou et al. applied a chemically reduced GO modified glassy carbon (CR-GO/GC) electrode for the DNA sensing [53], and showed enhanced electron transfer kinetics compared to graphite-modified glassy carbon (graphite/GC) and glassy carbon (GC) electrodes, thus demonstrating the improvement and robustness of CR-GO as an advanced carbon electrode material for electrochemical and biological sensing. Balapanuru et al. synthesized a charge-transfer complex composed of GO and pyrene dye PNPB, which exhibited a highly selective and rapid detection of DNA in biological mixtures which may also contain RNA, proteins, and glucose [54]. This is due to the formation of an ionic complex between DNA and PNP^+^ on GO, which switches on the fluorescence, as shown in Figure 5A. The other biomolecular species cannot remove PNP^+^ from GO due to the π–π stacking effect, thus quenching the fluorescence. Stine et al. employed nanometer-thick layers of reduced GO (rGO) to covalently attach with single-stranded DNA (ssDNA), and formed a field-effect transistor (FET) device to implement sensitive, real-time, and label-free detection of DNA hybridization [55]. Large-area deposition of rGO films and incorporation of reference sensors contributed to the improvement of detection specificity reported in their work, while the limit of detection for this rGO FET compared favorably with other types of label-free detection platforms, such as surface plasmon resonance (SPR) and nanowire devices. Wang et al. designed an aptamer—carboxyfluorescein (FAM)/GO nanosheet (GO-nS) complex to investigate DNA and protein probing in living cells, and revealed dramatic protection, delivery, sensing, and intracellular tracking capabilities of GO−nS [56]. Noncovalent binding between GO−nS and DNA strands indicated that GO−nS can serve as a good protector and an efficient cargo for cellular delivery of genes. Liu et al. utilized GO as a functional matrix to develop fluorescent sensors for amplified and multiplexed detection of DNA and aptamers [57], as shown in Figure 5B. Based on the specific interaction between DNA constructs and GO, they also implemented the activation of the “OR” and “AND” logic gates for the developed biosensing platform. Qian et al. developed a fluorescent sensing platform for DNA detection based on the regulation of interaction between GO and graphene quantum dots (GQD) [58], as shown in Figure 5C. The platform can distinguish the complementary and mismatched DNA sequences with high sensitivity, good reproducibility, and excellent biocompatibility; thus it may promote the application of carbon-based nanomaterials in effective immunoassays.

It is important to note that the oxygen concentration in GO may vary considerably based on different synthesis protocols or procedures, which could potentially influence the DNA-sensing effect. Quite a few investigations have been conducted to quantify this GO compositional factor. Hong et al. noticed that the oxidation level of GO has a strong impact on the binding interaction to ssDNA and the fluorescence-quenching ability [59]. They discovered that the less-oxidized GO can bind more strongly to ssDNA and quench the fluorescence more effectively than the more-oxidized GO, and the detection sensitivity in serum is much higher than that in Tris-HCl buffer, indicating a suitable application of DNA sensing by GO in biological fluids. Lu et al. pointed out that the preparation condition of GO, more specifically, the further reduction step which decreases the oxygen content in GO, can considerably affect the DNA-sensing efficiency [60]. They systemically compared the GO and reduced GO samples in DNA sensing and found that GO presented a higher signal enhancement and faster signaling kinetics, while rGO absorbed DNA more tightly, and exhibited a greater resistance to the desorption induced by temperature, pH, urea, and organic solvents. Wang et al. investigated the supramolecular interactions of aggregation-induced emission (AIE) probes and GO on DNA sensing [61]. They demonstrated that AIE probes with short alkyl chains manifest higher binding affinity with ssDNA and GO with a lower oxidation degree exhibits stronger binding interactions to ssDNA and greater fluorescence quenching efficiency.

Considerable efforts have also made to explain and clarify the mechanism that govern the GO biosensing for DNA detection, force generation, and nonspecific interactions. Liu et al. reported a mechanism of DNA sensing on graphene oxide [62]. They discovered a substantial fluorescence enhancement by exposing a fluorophore-labeled probe DNA, which was preabsorbed on GO, to the complementary DNA. Subsequently, they quantitatively demonstrated that the enhancement of DNA hybridization in the presence of GO results from the displacement of probe DNA into the solution for hybridization in response to the large adsorption energy difference between the probe DNA and its complementary DNA, but not the Langmuir−Hinshelwood mechanism (hybridization on GO, then desorption to solution) or the Eley−Rideal mechanism (direct hybridization in solution without complementary DNA adsorbed on GO). Lu et al. compared GO with the other 2D materials, molybdenum disulfide (MoS_2_) and tungsten disulfide (WS_2_) for DNA sensing [63], and explained the difference in surface forces for DNA adsorption based on the chemical structures of three biosensors, i.e., GO absorbs DNA mainly by hydrogen bonding and π–π stacking, while MoS_2_ and WS_2_ absorb DNA primarily by using of van der Waals force. Liu et al. conjugated GO with the nucleobases of DNA, while screening metal oxide nanoparticles to interact with the phosphate backbone of DNA, so as to reach highly sensitive DNA detection in serum and avoid the non-specific DNA displacement induced by proteins [64]. They reported that cobalt oxide (CoO) presents nearly full resistance to protein-induced DNA displacement, while nickel oxide (NiO) shows the best detection limit for DNA sensing.

The exceptional properties of GO biosensors make them an ideal choice for DNA sensing with high precision, selectivity, and sensitivity. Many types of GO biosensors in combination with glassy carbon, organic dyes, aptamer, and quantum dots have been reported to achieve the above goals with progressive improvement. Meanwhile, the oxidization level of GO serves as an important factor in affecting DNA-sensing efficiency. More specifically, the more reduced GO presents stronger binding with DNA strands and greater fluorescence quenching efficiency. Finally, the displacement of probe DNA, hydrogen bonding and π–π stacking, and nonspecific interaction induced by protein, are unveiled as generic mechanisms that govern GO biosensing for DNA detection.

## 5. Pathogen Detection Enabled by Functional Graphene 

The presence of various oxygen-containing functional groups on the GO surface provides good dispersibility and favorable binding sites for functionalization in designing high-quality biosensors for pathogen detection [65]. Analytes get attached to the GO surface due to the presence of polar groups such as hydroxyls, carboxyls, and epoxides mainly through electrostatic interactions providing a variety of interaction options for bonding [7]. Similarly, partially reduced GO(p-rGO) or rGO interact with the various biomarkers such as protein and DNA through van der Waals interaction [66]. In this way, GO offers stronger, better, multiple adsorption capacities by GO sheets which could influence the chemical bonds of pathogen body structures and be able to show improved sensing performance in various detection techniques (Figure 2). 

Among graphene derivatives, GO has been found to have the most active antibacterial activity [67]. Liu et al. developed an innovative sensing antimicrobial mechanism to trap bacteria using graphene nanosheets. The oxidative stress produced by graphene nanomaterial sheets captured bacteria and ruptured their membranes, reducing the metabolic rate of the bacteria. Wu et al. reported on the possible antimicrobial mechanisms of GO in tackling bacteria through (i) inducing cellular trauma with the sharp edges of the nanomaterial; (ii) oxidative stress caused by the generation of superoxides with treatment of graphene nanomaterials; and (iii) wrapping or trapping the bacteria, and limiting the physical movement and metabolism of the bacteria [11]. They demonstrated GO as a potential antimicrobial nanomaterial for effectively controlling multidrug resistant (MDR) pathogens such as *Klebsiella pneumoniae* (Kp), *Escherichia coli* (E. coli) and *P. aeruginosa* (Pa) for in vivo and in vitro studies. They showed that GO inhibited the growth and killing of Kp in macrophage and mouse models after GO solution were introduced with harvested bacterial suspension for 2 h at 37 °C and results were recorded. Researchers also explored the electrochemical properties of GO for sensing various biomolecules. Tiwari et al. developed a nucleic acid sensor using GO-modified iron oxide–chitosan hybrid nanocomposite (GIOCh) film for detection of *Escherichia coli* O157:H7 (*E. coli*) [68]. The pDNA immobilized onto the GIOCh/ITO sensor exhibited high sensitivity of 1 × 10^−14^ M. Researchers also fabricated GO-based devices to clean the environment using pathogen-like hyphae fungus to fabricate a mechanically stable thin film sensor. Zhang et al. developed highly flexible porous film for dye removal by graphene oxide–fungus interaction. They designed a flow-through adsorption device using GO and fungus hyphae which absorbed the target dye pollutant to clean the environment [69].

Virus infection is a global phenomenon, and the COVID-19 pandemic has caused havoc by infecting and killing almost 1.7 million people worldwide between late 2019 and mid-2021. Therefore, we require more robust and sensitive early detection systems to control the global pandemic caused by deadly viruses such as the corona virus. One of the earliest works for pathogen detection using GO was led by Lu et al., who demonstrated water-soluble GO as a new platform for the sensitive and selective detection of DNA and proteins [70]. They explored the fluorescence quenching properties of GO in DNA biosensing using a fluorescein-based dye. Similarly, Jung et al. reported on a simple, highly sensitive and selective GO-based biosensor platform for detecting rotaviruses [71]. The detection occurred by GO photoluminescence quenching induced by fluorescence resonance energy transfer (FRET) between GO sheets and AuNPs. The high affinity between gold nanoparticles and the amino functional groups of the DNA nucleotides provided a selective attachment of target cells of the rotavirus to the GO sheets. This interaction resulted in detection of rotavirus cells due to reduction in the fluorescence quenching of GO.

One interesting work was reported by Song et al. who developed a novel GO-based label-free method to capture and disinfect environmental viruses (enteric EV71 and H9N2) [72]. They demonstrated that GO interacted with the membrane of the virus to extract the viral RNA and finally destroyed the virus to prevent further transmission in the environment. Under optimal temperature with prolonged exposed time, GO was able to denature the protein structure of the virus by breaking the chemical bonds. This novel method showed a simple method of reducing the risk of infection with minimized environmental contamination and reduced time, processing, and cost.

**Figure 6:**
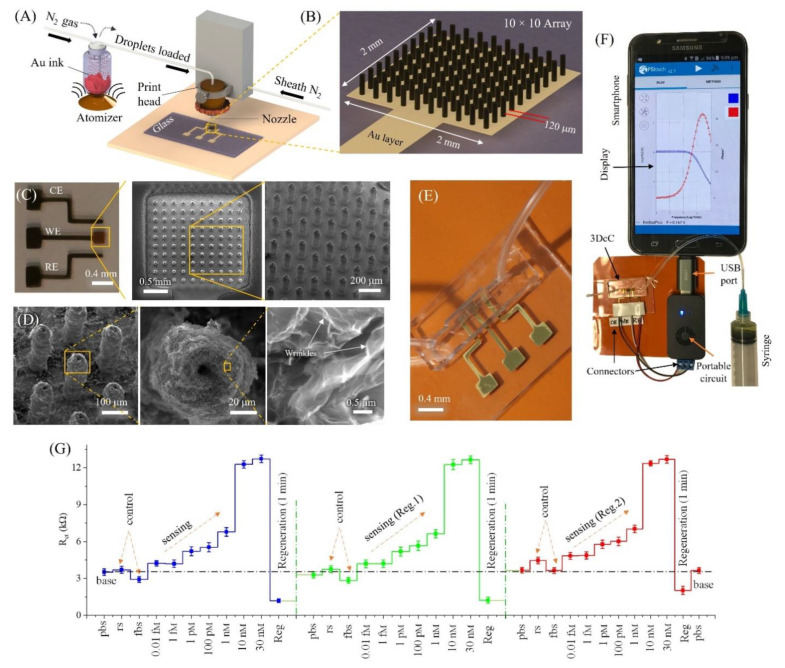
Graphene-based 3D printed sensor for detecting COVID-19 antibodies in seconds. (**A**) A schematic of the aerosol-Jet 3D nanoparticles printer. Gold nanoparticle (size ~4 nm) ink was loaded in an ultrasound automizer and created aerosol droplets by applying ultrasound force. A carrier gas (N_2_) was used to carry aerosolized droplets to the printed head and a sheath gas (N_2_) was further applied to aerodynamically focus the aerosol ink before printing on the substrate. A 10 × 10 array was created using this printer wherein an individual pillar consists of multiple rings that are stacked together. (**B**) The printed gold pillar was thermally sintered. (**C**) SEM images showed the top of the printed array. (**D**) The micro-textured surface of gold was decorated with rGO nanosheets to hold COVID-19 spike S1 protein (antigen). (**E**) A photograph of complete device integrated into a microfluidic cell and (**F**) sensor integration with a smartphone platform. (**G**) Sensing results of COVID-19 S1 antibodies wherein the charge transfer resistances (R_ct_) were plotted against the concentration ranging from 0.01 fM to 30 nM. Rabbit serum (rs), fetal bovine serum (fbs), and phosphate buffer saline (pbs) were taken as control biofluids. Sensing shown in blue panel was produced without sensor regeneration, after first regeneration using a low pH solution shown in green panel. The red panel demonstrated the sensor response with second regeneration. Reprinted with permission from ref. [73]. Copyright 2021 Wiley Online Library.

GO-based microfluidic immunosensors are becoming attractive alternatives to traditional pathogen-detection techniques such as ELISA, cell culture, and rt-PCR for better clinical tests due to rapid diagnosis, cost effectiveness, easy application, and high reproducibility. In the current scenario, we require highly sensitive, rapid, and early detection tools for quick diagnosis of highly infectious disease such as COVID-19 and the Zika and Ebola viruses. Figure 6 shows an innovative immunosensor chip using 3D nanoprinting of three-dimensional electrodes of gold nanopillars known as the ‘3D-printed COVID-19 test chip (3DcC)’ which were coated with nanoflakes of reduced graphene-oxide (rGO) [73]. This device was created using an aerosol-jet 3D nanoparticle printer wherein a 10 × 10 micropillar array was created by layer-by-layer printing (Figure 6A,B). The array was coated with rGO nanoflakes and functionalized with spike S1 antigens of SARS-CoV-2 (His Tag) enabled by EDC-NHS chemistry. Figure 6 C, D shows the SEM images of micro-textures of printed micropillar array. An optical image of this device is shown in Figure 6E. The sensor was designed with two different spike antigens such as S1 and RBD receptor-binding domain (RBD) specific to COVID-19 antibodies (immunoglobin; IgG). This sensor has an interface with a smartphone-based readout (Figure 6F) and showed 9-time regeneration ability to detect COVID-19 antibodies. The sensor detected COVID-19 antibodies within 10 seconds via an electrochemical transduction mechanism. Sensing results of this device for S1 antibodies are shown in Figure 6G. In addition, this rapid test enabled by rGO has the potential to investigate the immune dynamic of the COVID-19 patients at their different stages of infection which is important keeping in mind the various difficulties with COVID-19 testing [74]. We believe that merging the manufacturing of graphene-based sensitive biosensors with wearable technology and the internet of things (IoT) can introduce the next generation of innovative technology for better and economic health care. We discuss future biosensor technology using graphene and its derivative using advanced manufacturing in the next section.

## 6. Future Perspectives—3D Printed Graphene-Based Biosensors

Graphene-based nanomaterials as transducing biosensing materials have shown great promise due to their large surface area, compatibility with biomolecules, electron transfer rate, and ability to immobilize a variety of different biomolecules. Although graphene-based materials are excellent electrode materials for biosensing applications, their application in industrial products is limited. In addition, manufacturing of compact biosensors at low cost is an important factor in biosensor technology since it involves development of reproducible, robust, and reliable sensors with high sensitivity, specificity, and low limit-of-detection. Traditional manufacturing processes such as screen printing of graphene electrodes provided two-dimensional surfaces which limited the performance of devices. So far, many techniques have been explored to produce graphene and graphene-derived nanomaterials such as thermal decomposition [75], mechanical exfoliation [8], the hummer method [76], and chemical vapor deposition [77]. GO/rGO synthesis using wet chemical methods is a tedious and time-consuming process which requires a series of oxidation/reduction steps, repeated washings, and centrifugation resulting in uncontrolled oxygen functionalities at the sheets. In addition, GO/rGO sheets in liquid solutions tend to restack to generate graphite [78]. After synthesis of graphene using these methods, their manufacturing in terms of electrochemical electrodes is still challenging. One of the methods, electrodeposition of graphene, was utilized to fabricate graphene electrodes [79]. However, the mass production of graphene electrodes using electrodeposition is a major concern due to structural integrity issues. 

Recently, Ghanam et al., introduced a simple, scalable technique called laser-scribed graphene (LSG) which employed a laser beam in order to convert carbon or polymer precursor films into three-dimensional graphene electrodes without using any lithographic mask [80]. This mask-free manufacturing method of graphene electrodes offered film uniformity, mass production, and multilayered combination of graphene sheets with high porosity and functional groups which are excellent features to construct sensitive biosensor devices [80]. However, laser resolution for patterning graphene, laser quality and speed, precise control of z-distance, and the fragile nature of graphene structures leading to lower conductivity of electrodes are still impediments to mass-scale adoption. Technology for manufacturing graphene-based biosensors at the industry level is thus still in its early stages.

Owing to the excellent electrical and electrochemical properties of graphene and their integration into flexible devices, graphene-based materials can provide versatile neural recording probes which help to solve several problems in neural interface design [81]. Graphene provided increase adhesion, biocompatibility, and good viability with cell cultures which can lead to the next generation of flexible neural implants [81]. For in vivo brain activity recording, graphene showed good signal-to-noise ratio due to implantation of 3D graphene with high specific surface area, high porosity, and high spatial resolution [82]. Further, high mobility of charge carrier, high transconductance, and low intrinsic noise of graphene field-effect transistors allowed them to detect action potentials of electrically active cells in vitro and in brain activity [83,84]. In addition, graphene and its derivatives are an excellent choice of materials which can be modified with specific bio-recognition molecules for in situ biosensing of many neuromodulators and neurotransmitters including dopamine, histamine, and glutamate in the central and peripheral nervous systems.

The fourth industry revolution (i.e., Industry 4.0) is an innovative approach and shows the massive capability of manufacturing biomedical devices including biosensors [85]. In recent years, advanced manufacturing (i.e., 3D printing) has revolutionized the manufacturing of biosensing structures with complex and customized features [86]. This enables three-dimensional layer-by-layer printing with well-defined features at micro and sub micrometer scale, and complex architectures of nanomaterials, polymers, and their composites resulting in rapid prototypes in a controlled manner [87]. Compared to traditional subtractive-based manufacturing processes such as drilling, milling, sawing, and broaching, additive manufacturing can directly print three-dimensional parts through the sequential layer stacking of materials, thus enabling enormous possibilities for rapid prototyping and customized devices [88]. Note that 3D printing is a maskless and non-lithographic process that can be performed by a click from the computer-aided-design file [89] and does not require a clean room. The resolution of printing is dependent on the methods of printing. The high-resolution capabilities of 3D printing create new manufacturing opportunities for biosensor devices. Potential merits of 3D printing are design freedom, flexibility, customizability, material combinations, and high sustainability. 3D printing can be categorized mainly by seven different types such as material extrusion, vat photopolymerization, powder bed fusion, material jetting, binder jetting, sheet lamination, and direct energy deposition. Since the invention of stereolithography (SLA) in 1986 by Chuck Hull [90], there have been many methods of 3D printing such as digital light processing (DLP), materials jetting (MJ), fused deposition modeling (FDM), direct ink writing (DIW), selective laser sintering (SLS), multi-photon polymerization (MPP), and aerosol jet (AJ) 3D printing. Printing resolution can vary according to the type of 3D printer [89,91]. Among all techniques, extrusion-based printing is an attractive method for printing graphene due to its easy printing process and the convenience of transferring graphene solution in the solution form to the printing machine. For example, DIW is an extrusion-based 3D printing which can deposit layer-by-layer of liquid graphene ink that quickly solidifies upon extrusion resulting in 3D parts.

**Figure 7:**
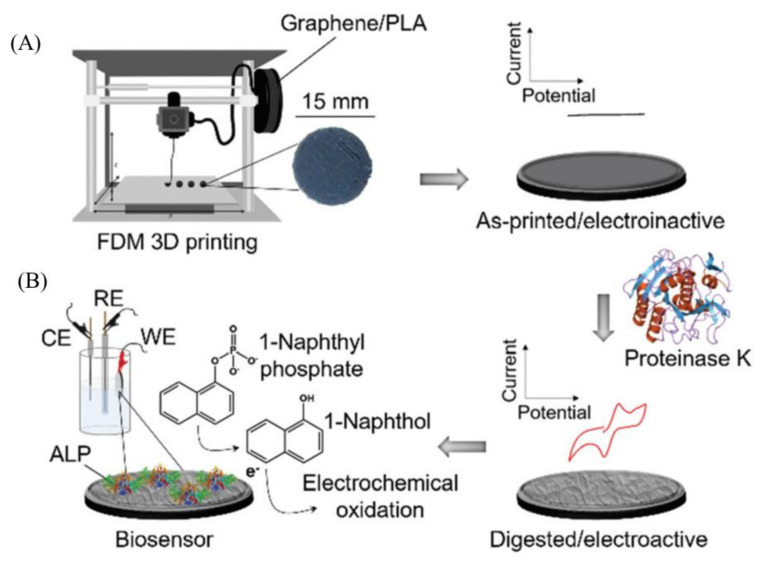
(**A**) Schematic of a fused deposition modelling (FDM) printer. A coin-shaped electrode was made of graphene and PLA composite filament 3D-printed with a FDM printer. The printed electrode was electrochemically irresponsive for the ferro/ferricyanide redox marker, however, after being modified with proteinase K-mediated PLA the electrode became eroded and electrochemically active. (**B**) The 3D printed electrode was modified with alkaline phosphatase (ALP) enzyme on the graphene surface, which acted as a catalytic electrode towards the conversion of 1-naphthyl phosphate into 1-naphtho [93]. Reprinted with permission from ref. [93]. Copyright 2019 RSC.

Three-dimensional platforms of graphene electrodes have enhanced biosensing performance compared to two-dimensional electrodes in terms of sensitivity, limit-of-detection, and selectivity indicating their importance in next-generation biosensor development. Jakus et al., created a 3D printed scaffold structure consisting of graphene sheets and a biocompatible material of polylactide-co-glycolide with minimum features of 100 μm and improved electrical conductivity [92]. Polylactic acid’s (PLA) biodegradability has been exploited to fabricate 3D-printed graphene/PLA surfaces selectively and reproducibly in the detection of 1-naphthol in aqueous solutions using alkaline phosphatase enzyme(ALP) [93]. This is a single-step fabrication process enabled by FDM for the development of a 3D biosensor where proteinase K-mediated partial digestion of the PLA filament results in the exposure of active graphene edges. The ALP is then adsorped on the exposed surface to create the sensing electrode (Figure 7). Further, another 3D-printed graphene electrode was employed to create an enzymatic biosensor [94]. In this sensor, the graphene-PLA electrode was functionalized with horseradish peroxidase enzyme which enabled direct electron transfer for hydrogen peroxide (H_2_O_2_) in human serum and had a stable response after 7 days of incubation. This sensor did not require any mediator to detect H_2_O_2_, and thus realized a third-generation biosensor [94]. However, due to the presence of binders and other surfactants, the graphene-based 3D printed electrode manufacturing showed poor performance. High levels of binder in graphene inks may lead to problems of nozzle clogging due to high viscosity while low levels of binder may result in film cracking. Solvent-assisted graphene nano-ink such as graphene dispersed in dimethylformamide solvent can be used to print 3D structures of graphene to improve the device performance. A 3D printed graphene-PLA electrode was treated with dimethylformamide (DMF) before the immobilization of enzyme by enabling crosslinking of glutaraldehyde [95]. This sensor was utilized for simultaneous quantification of uric acid and nitrite in human urine and saliva, respectively. Silva et al. [96] have also described sequential chemical treatment to produce rGO within 3D-printed PLA, where a rGO-modified electrode produced after washing with NaBH_4_ showed increased current density for the redox probe ferrocene-methanol in contrast to PLA surface treated only by DMF. This is attributed to a better reorganization of the rGO surface with reduction in defects and enhanced exposure of electroactive sites validated using characterization through SEM, AFM, and Raman spectroscopy. The sensor produced encouraging results in determining catechol in natural water samples.

A review paper was recently published on additive-manufactured-based electrochemical sensors [97]. That review summarized the possibilities of selective laser melting and fused deposition molding (FDM)-based 3D printed electrodes in the area of electrochemical sensors systems. However, until now, only a few biosensors have been reported based on 3D printed graphene electrodes. Table 1 summarizes the most-cited graphene oxide based biosensors utilizing 2D and 3D sensor structures. Further research is needed to create 3D graphene biosensors at a large scale and integrate them with wearable technology and the internet of things (IoT) for health monitoring. In the future, additive manufacturing approaches will be the next manufacturing technology for creating biosensor devices with a range of sensing structures and electrodes. However, this is still at an early stage, and the cost of manufacturing is still a major concern. We are expecting that the next generation of 3D printing technology can reduce the cost of manufacturing significantly and will allow fabrication in a convenient way by reducing instrument costs for large-scale production.

## 7. Conclusions

In summary, functional graphene and its derivatives are exciting materials in the world of nanotechnology. The excellent properties of graphene have revolutionized the development of electrochemical, fluorescence, and optical biosensors for point-of-care health diagnostics. Continuous and growing applications of graphene are replacing the current technologies and opening a new direction of biomedical sensors with high performance. In this review, we covered the application of graphene and its derivatives for the detection of a range of biomarkers including toxins, antibodies, pathogens, and DNA. As the functionality of the graphene can be tuned for a specific application of biomarker sensing, we covered a set of examples of biosensing with different modalities. We also critically reviewed the fundamentals of functional GO and rGO towards DNA sensing, enzymatic biosensors, monitoring of pathogens, and immunosensing. It is noted that GO is more amenable to mass-scale production from the viewpoint of cost optimization and manufacturability when compared to graphene. GO can be easily used in composites with other polymers to produce electrochemically active, mechanically robust, functional transducers for biosensors. GO-based platforms have been successfully used in electrochemical-, FET- and surface-plasmon-resonance-based biosensors. Moreover, rGO offers the advantage of various functionalization schemes for the recognition of elements of biosensors and thus provides multidimensional advantages compared to other biosensing transducer materials. In addition, we described the possibilities of the next-generation graphene-based biosensors using advanced manufacturing such as 3D printing. It is noted that the production of high-quality defect-free graphene sheets and their use in industrial manufacturing of biosensors are still limited. This will need to be overcome for scaling up the production of graphene. We are expecting that future research would address these challenges for the commercial production of graphene at a low cost. In the coming years, we believe that the additive manufacturing of graphene combined with IoT, and wearable technology can potentially transform the market of traditional biosensor technology for health monitoring. 

## Figures and Tables

**Table 1:** Biosensors based on graphene oxide by enabling 3D printing and 2D traditional manufacturing. (FET, field effect transistor; BSA, bovine serum albumin; PLA, polylactic acid; DPV, differential pulse voltammetry; EIS, electrochemical impedance spectroscopy).

Transducer Materials	Modalities	Type of Sensor	Analytes	Ranges	LOD	Refs.
GO on Gold Electrode (2D)	SERS	Immunosensor	Cancer proteins and cells	1 fg/mL to 10 pg/mL in PBS	1 fg/mL	Reza et al. [14]
Carboxy-Functionalized GO (2D)	SPR	Immunosensor	Anti-BSA	0.01–100 pg/mL	0.01 pg/mL	Chiu et al. [98]
Inkjet-Printed GO/Pentacene (3D)	FET	DNA sensor	Artificial DNA	0.1–100 pmoles/µL	0.1 pM	Lee at al. [99]
AuNP-Graphene oxide (2D)	Chronoamperometry	DNA sensor	Breast cancer biomarker ERBB2	0.37–10 nM	0.16 nM	Saeed e al. [100]
Screen-Printed GO film (2D)	EIS	Immunosensor	Influenza A virus	10 ng/mL to 10 μg/mL	10 ng/mL	Kinnamon et al. [101]
3D-Printed Au- rGO Array (3D)	EIS	Immunosensor	COVID-19 Antibody	0.01 fM–30 nM in PBS	2.8 fM	Ali et al. [73]
3D-printed rGO/PLA Electrode (3D)	DPV	Enzymatic	Phenolic compound catechol	30–700 μmol/L in PBS	0.26 μmol/L	Silva et al. [96]
Au-GO (2D)	Voltammetry	Enzymatic	Cholesterol	0.01 μg/mL to 5000 μg/mL	0.001μg/mL	Huang et al. [33]
